# The design of a point of care FET biosensor to detect and screen COVID-19

**DOI:** 10.1038/s41598-023-31679-5

**Published:** 2023-03-18

**Authors:** Nisreen Alnaji, Asma Wasfi, Falah Awwad

**Affiliations:** 1 Department of Electrical and Communication Engineering, College of Engineering, United Arab Emirates University, P. O. Box 15551, Al Ain, United Arab Emirates; 2 Zayed Center for Health Sciences, United Arab Emirates University, Al Ain, United Arab Emirates

**Keywords:** Engineering, Nanoscience and technology

## Abstract

Graphene field effect transistor (FET) biosensors have attracted huge attention in the point-of-care and accurate detection. With the recent spread of the new emerging severe acute respiratory syndrome coronavirus 2 (SARS-CoV-2), the need for rapid, and accurate detection & screening tools is arising. Employing these easy-to-handle sensors can offer cheap, rapid, and accurate detection. Herein, we propose the design of a reduced graphene oxide (rGO) FET biosensor for the detection of SARS-CoV-2. The main objective of this work is to detect the SARS-CoV-2 spike protein antigen on spot selectively and rapidly. The sensor consists of rGO channel, a pair of golden electrodes, and a gate underneath the channel. The channel is functionalized with COVID-19 spike protein antibodies to achieve selectivity, and with metal nanoparticles (MNPs) such as copper and silver to enhance the bio-sensing performance. The designed sensor successfully detects the SARS-CoV-2 spike protein and shows singular electrical behavior for detection. The semi-empirical modeling approach combined with none-equilibrium Green’s function were used to study the electronic transport properties of the rGO-FET biosensor before and after the addition of the target molecules. The sensor’s selectivity is also tested against other viruses. This study provides a promising guide for future practical fabrication.

## Introduction

SARS-CoV-2 or what’s known as severe acute respiratory syndrome coronavirus 2 was and still the center of extensive research since its outbreak. Different early and accurate detection techniques have been proposed to aid in the viral containment such as reverse transcription polymerase chain reaction (RT-PCR)^1^, radiologic photography^2^, chest CT^3–5^, artificial intelligence (AI)^6–8^, reverse transcription loop-mediated isothermal amplification (RT-LAMP)^9^ and clustered regularly interspaced short palindromic repeats (CRISPR-Cas)^10^.

Biosensors have always been successful tools in detecting viral infections. They have been used to detect different viruses such as Ebola^11,12^, Hepatitis A^13^, Influenza A^14,15^, Zika^16^ and Hanta^17^. Indisputably, biosensors played a major role in the detection of SARS-CoV-2 also. For instance, FET with the help of 2D semiconducting materials were utilized to detect SARS-CoV-2. It can offer a rapid detection solution. Fathi-Hafshejani et al.^18^ used SARS-CoV-2-antibody functionalized semiconducting transition metal dichalcogenide (TMDC) WSe2 monolayers as the detection platform and reached a limit of detection (LOD) of 25 fg/μL. Seo et al.’s group^19^ introduced graphene based FET to detect the virus. After preparation of the graphene layer, Au/Cr electrodes were etched. Then, the graphene channel was coated with antibodies against the viral spike protein. The sensor reached a LOD’s of 1.6 × 101 pfu/mL in culture medium. However, according to Clemens et al.^20^ study, field effect-based biosensors (BioFETs) suffer from two major challenges. First, getting an accurate accepted results is based on the self-consistent calculations that must include all the charges of the system. Second, BioFETs arrangements have multi-scale nature i.e., the biomolecules are on the angstrom scale while the device can reach to micrometer range. Moreover, FETs can also be designed as ZnO nanorods which have shown a remarkable performance in terms of stability^21^, that made it a great candidate to be used for the detection of Prostate Specific Antigen (PSA)^22^ and Hepatitis B surface antigen^23^. Another interesting application using ZnO was reported by Chakraborty’s team, they employed the ZnO to create a thin film transistor (TFT) to detect Hepatitis B surface antigen the sensor was used to measure and enhance the reliability of bio-targets detection^24^. Nanomaterials based sensors and assays also played a major role in the detection race. Huang et al.^25^ designed a AuNPs decorated lateral flow strips to detect the produced IgM antibodies against SARS-CoV-2. Additionally, Mahari’s group^26^ designed AuNPs decorated, fluorine doped and nCovid-19 monoclonal antibody (SARS-CoV-2 Ab) coated tin oxide electrode (FTO) as a potentiostat sensor to sense any change in electrical conductivity, and then compared its performance with an in-house built eCovSens potentiostat. The LOD’s of 90 fM and 120 fM were recorded for eCovSens and potentiostat, respectively. The applicability of another interesting nanomaterial named Quantum Dots (QDs) is still being evaluated with a promising expected performance according to Sultan et al.’s review^27^. Plasmonic photothermal (PPT) effect along with localized surface plasmon resonance (LSPR) was suggested. The production of PPT heat on the complementary DNA functionalized, nano-sized gold islands give rise to the in-situ hybridization temperature and leads to specific differentiation between couple of similar genetic sequences. This technique is very sensitive, rapid and reach a LOD of 0.22 pM^28^. Zhang et al.^29^ proposed a surface-enhanced raman scattering (SERS) based biosensor to detect SARS-CoV-2 in untreated saliva. The sensitivity and reproducibility were guaranteed by using three-phase (oil/water/oil) liquid–liquid system which make up two layers of AuNPs.

Carbon which is the primary material of all living structures can be found in different arrangements around us, for example it can be spherical forming fullerenes, or rolled along specific direction giving carbon nanotubes. It can also be in a three-dimensional structure producing weakly bonded graphene layers known as graphite and it may be found in a honeycomb-like arrangement revealing graphene^30^. Unlike most of semiconductors which focus on the zero-momentum point Г when studying electronic properties, K and K′ points are the focus for graphene. This is not the only difference between graphene and conventional semiconductors, there are a few interesting differences to be mentioned. Firstly, energy band gap, graphene is gapless while other semiconductors have finite gaps. Secondly, graphene has a chiral, linear dispersion relation, however semiconductors have quadratic one. Thirdly graphene is much thinner compared to 2D electron gas, in fact it is the thinnest material found until now. Finally, graphene has finite minimum conductivity, irrespective of diminishing fermi energy, which is a key point to be noted when designing FETs^31^.

Graphene at its raw state may not be suitable for electronics applications. The gapless nature of graphene prevents it from being used as a channel material in FETs. The presence of two identical carbon atoms within the unit cell causes the absence of any energy gap. Thus, to open a bandgap the planar symmetry should be broken by structural or chemical modifications^32^. Graphene’s bandgap can be tailored using several techniques, such as, fabricating graphene nanoribbons^33^, applying a vertical electrical field on bilayer graphene^34,35^, strain^36^, doping^37–41^ and the reduction of GO^42–44^.

Reduced graphene oxide (rGO) as a derivative of graphene has several unique electrical properties. The existing functional groups on its surface pave the way for increased control and tuning of its electronic behavior. There are various advantages offered by controlling the amount of oxygen functional groups in rGO, for example the reduction of GO in hydroiodic acid, causes a noticeable improvement in the productivity^45^. Moreover, literature has also investigated the dependence of transparency and conductivity on the GO degree of reduction and its films’ thickness^46^. Additionally, the degree of oxygen functional groups elimination was again the reason of decreasing the rGO interlayers spacing’s^47^. In addition to the aforementioned benefits, oxygen functional groups are also considered as active binding sites for the desired molecules such as nanoparticles or antibodies which allow further engineering of rGO sheets^47,48^.

In this work we introduce the design and simulation of a point-of-care FET based biosensor for the rapid and selective detection of SARS-CoV-2. The main aim of the current study is to closely observe the transport electronic reaction (i.e., transmission spectrum, output and transfer curves, and current) of the FET-based biosensor against COVID-19 and other viruses (i.e., Rabies, and MERS). The research can be considered as a road map and first step before carrying on with practical fabrication. The designed sensor is expected to provide real-time, rapid and accurate detection results.

The sensor is composed of rGO channel, a pair of gold electrodes, and a gate underneath the channel. The rGO channel is coated with COVID-19 spike protein antibodies to achieve selectivity. Moreover, the biosensing performance and specificity are governed by decorating the sensor’s channel with metal nanoparticles (MNPs) such as, copper, and silver.

The employed semi-empirical approach was chosen based on its suitability for experimental data fitting, lower computational cost, and high accuracy^49^. This work is a proof of concept that the proposed FET-based biosensor has the ability to detect COVID-19 spike protein based on the singular electronic behavior found by the simulations performed.

The novelty in this paper is about investigating the effect of adding metal nanoparticles (i.e., silver, and copper) to enhance the biosensing performance of the designed rGO FET based biosensor. The results confirm that the addition of the metallic nanoparticles such as silver enhanced the detection signal (current variation). The addition of metallic nanoparticles can improve the FET sensitivity by increasing the surface area of the channel, which can result in a higher electron density and improved charge transfer^50^. The "plasmon-enhanced field-effect transistor (PEFET)" is a technique that increases the FET's sensitivity by adding metallic nanoparticles, and it has a significant potential for COVID-19 detection. In addition to increasing the surface area, the metallic nanoparticles can also enhance the sensitivity of the FET through the phenomenon of localized surface plasmon resonances (LSPRs).

The quantity of the target biomolecule that is adsorbed onto the surface of the FET channel determines the sensitivity of a FET biosensor. The surface area that is available for the adsorption of the target biomolecule is increased when metallic nanoparticles are introduced to the surface of a FET. This increase in surface area results in a higher density of target biomolecules adsorbed onto the FET channel, which in turn leads to a higher current detection signal.

## Materials and methods

### rGO FET design and configuration

**Figure 1:**
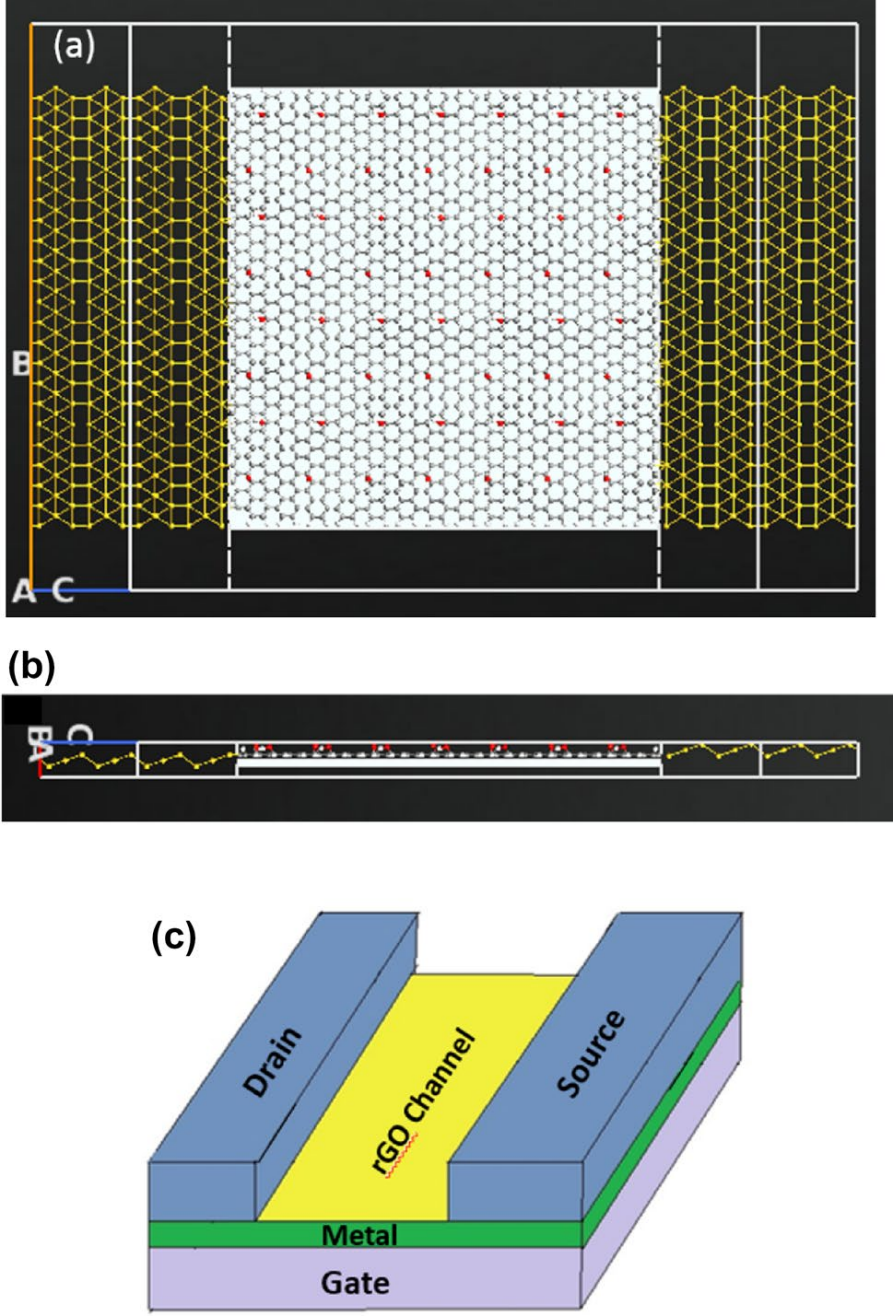
Schematic representation of rGO-FET composed of source, drain, channel, and gate underneath the channel (top view). The source and drain are made of gold and the gate consists of two layers: a dielectric layer and a metallic layer. (**a**) ATK-VNL top view (**b**) ATK-VNL side view (**c**) 3-D schematic diagram of the designed FET biosensor. (**a**) and (**b**) are produced by QuantumATK software (version 2018.06) while c) is produced by paint software (version 22H2).

According to literature the gate electrode can be placed either on top, back or top and back (dual structure) of the channel, the most suitable position for bio-sensing applications is the back gated since it allows the channel to be in full contact with the sensing medium^51,52^. The gate for the designed sensor is placed at the back of the channel but not touching it. The gate thickness is 1 Å and it consists of two layers: a dielectric layer of silicon dioxide with a relative dielectric K = 3.9 and a metallic layer. Top, side views and schematic 3D diagram of the full FET device is shown in Fig. 1. The designed electrodes are made of gold, with a length and width of 11.89 Å and 60.56 Å (1.189 nm, 6.056 nm) respectively. Dirichlet condition along C direction and Neumann boundary condition along A and B directions were applied on the electrodes when studying the device’s performance.

The transport drain current in the sensor’s channel is assumed to be following the below equation^53^:

1 $${I}_{d}=\frac{W}{L}\mu {C}_{ox}\left[\left({V}_{g}-{V}_{o}\right){V}_{ds}-\frac{{V}_{ds}^{2}}{2}\right]$$

where, W, L, µ, C_ox_, V_g_, V_o_ and V_ds_ are channel’s width and length, carrier mobility, geometrical capacitance, gate-source voltage, Dirac neutrality point and drain-source voltage respectively.

Moreover, the carrier mobility of the graphene FET depends mainly on the transconductance (g_m_) which is a very critical quantity to be studied for measuring sensor’s performance and is found by^53^:

2 $$\mu = \frac{{g}_{m}}{{V}_{ds}. {C}_{ox}}\cdot \frac{L}{W}$$

**Figure 2:**
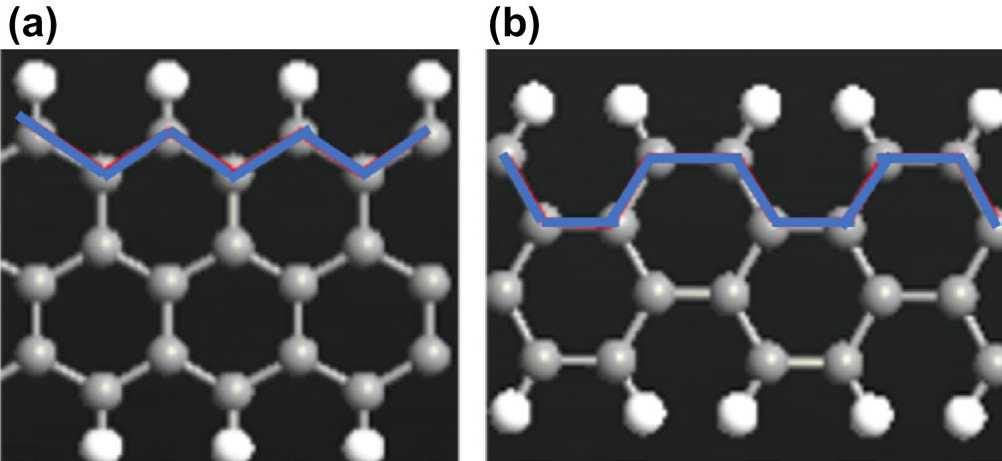
Graphene nanoribbons types. (**a**) Zigzag nanoribbon (**b**) armchair nanoribbon. Produced by QuantumATK software (version 2018.06).

The rGO channel was designed while considering the following critical points: Graphene nanoribbons can either have zigzag or armchair edges as shown in Fig. 2. The former one has metallic nature and cannot be used as a channel material while the latter can be considered as semiconductor since it is narrower than 10 nm^54,55^. Oxygen functional groups spread on the surface of rGO randomly. Studies have found four types of oxygen containing groups in graphene namely, hydroxyl (C–OH), epoxide (C–O–C), carboxyl (COOH), and carbonyl (C=O) with the former two being located at the basal plane and are responsible for most of the graphene’s unique electronic properties while the latter ones were found on the edges^56–60^. The channel designed here contains only hydroxyl and epoxide groups since carbonyl are not stable and converts into epoxide whereas carboxyl is only stable on defects sites which is not presented in our channel^43^. Channel dimensions were selected based on the viral spike protein size, the channel length is 6.124 nm, and the channel width is 5.896 nm. Channel functionalization basically constitute of three main components namely, MNPs, bio-recognition molecules and edges passivated with hydrogen. MNPs are employed here for two major reasons. First, since the surface of graphene is not chemically suitable for direct immobilization of biomolecules, metal nanoparticles (MNPs) are often used on rGO to enhance its bio-sensing performance and specificity^61–63^. Secondly, MNPs is used to avoid the immediate interaction between the rGO channel and the biomolecules since the immediate interaction can affect the performance^64^. Bio-recognition molecules are used to enhance the selectivity of the sensor and amplify its affinity. The rGO channel was coated with SARS-CoV-2 antibodies which are specific against the viral spike protein and known as probes^65,66^. The rGO channel edges were passivated with hydrogen. This step is crucial to saturate the dangling bonds, increase the edge-carbon stability, and effectively open a bandgap^67,68^.

**Figure 3:**
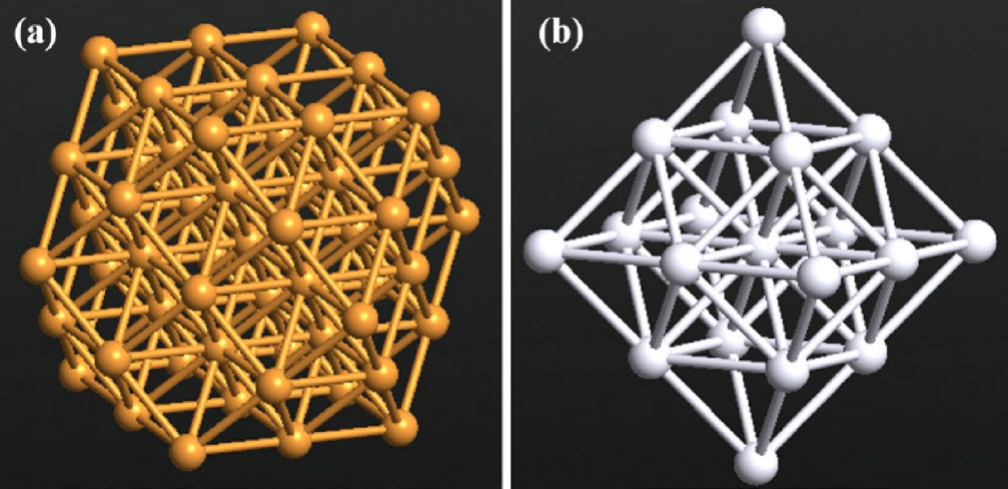
The added MNPs with 5 Å radii (**a**) copper (**b**) silver. Produced by QuantumATK software (version 2018.06).

Literature has reported that MNPs can be placed on hydroxyls or epoxies^48^. Copper and silver nanoparticles were added on the channel. Bare and MNPs decorated sensor were studied in terms of electrical current and selectivity. A special plug-in builder known as Wulff Constructor in the Quantumatk software was used to build the MNP’s. The radii of the added MNPs is 5 Å. The designed MNPs is shown in Fig. 3.

### SARS-CoV-2 spike protein

**Figure 4:**
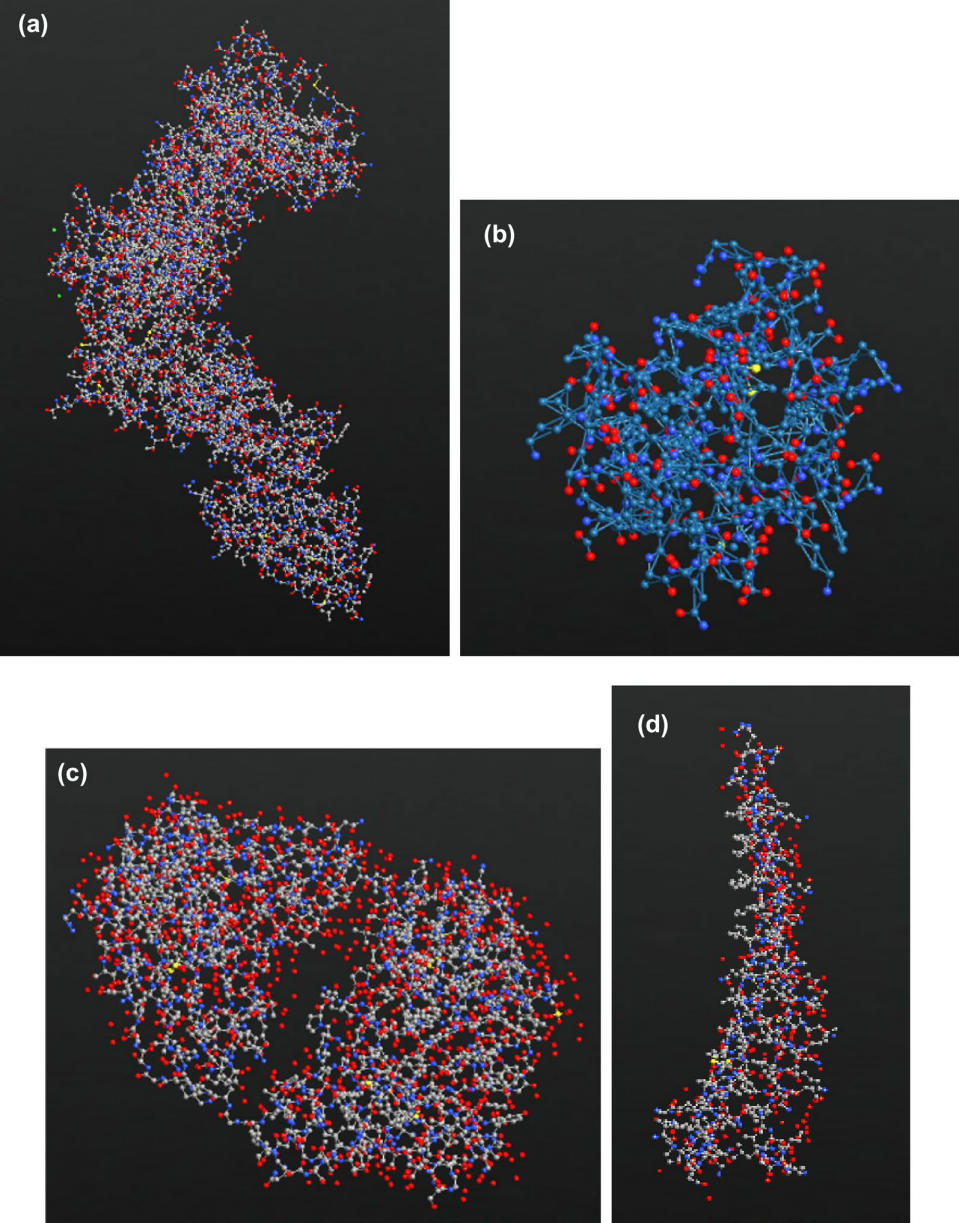
Different structures imported into the Quantumatk software (**a**) the imported viral spike protein (**b**) Rabies virus (**c**) anti SARS-CoV-2 human neutralizing antibody (**d**) MERS virus. Produced by QuantumATK software (version 2018.06).

**Figure 5:**
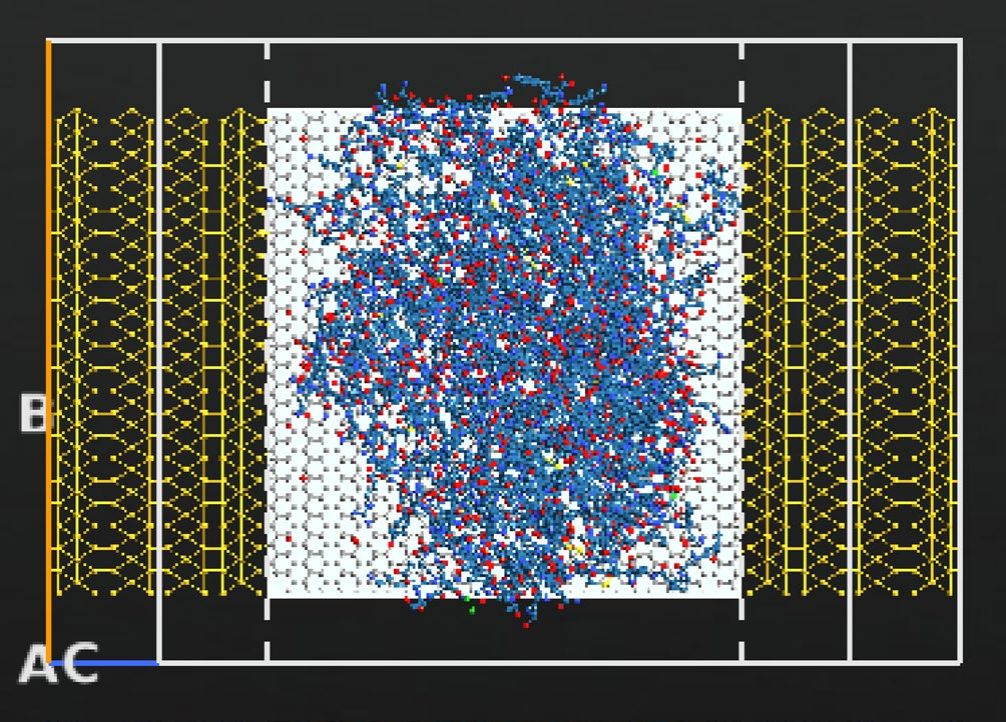
Top view of the designed biosensor with Cu MNPs, COVID-19 antibody and COVID-19 spike protein. Produced by QuantumATK software (version 2018.06).

SARS-CoV-2 spike protein is the target to be detected by the designed sensor, it was imported from an online protein data bank named RCSB Protein Data Bank^69,70^. The PDB ID of (Spike protein) is 2IEQ^71^, while the PDB ID of (SARS-CoV-2 virus spike receptor- binding domain complexed with a neutralizing antibody) is 7BZ5^72^. Figures 4a and 5 show the spike protein and the biosensor conjugated with MNPs, bio molecules, and spike protein, respectively. It is also worth mentioning that the spike protein is the main spot for many mutations, specifically in the N-domain, according to Helena’s group^73^, three strains are of concern which are B.1.1.7, B.1.351 and P.1/B.1.1.28.1 they have noticeable impacts on the transmissibility, treatment effectiveness and the reached level of immunity^73,74^. It is anticipated that since the target of our study is the spike protein normally any mutation in the same should be detected by our proposed sensor thanks to these mutations’ distinct atomic structure which will firmly result in singular electronic response for each one of them.

### Device testing

**Figure 6:**
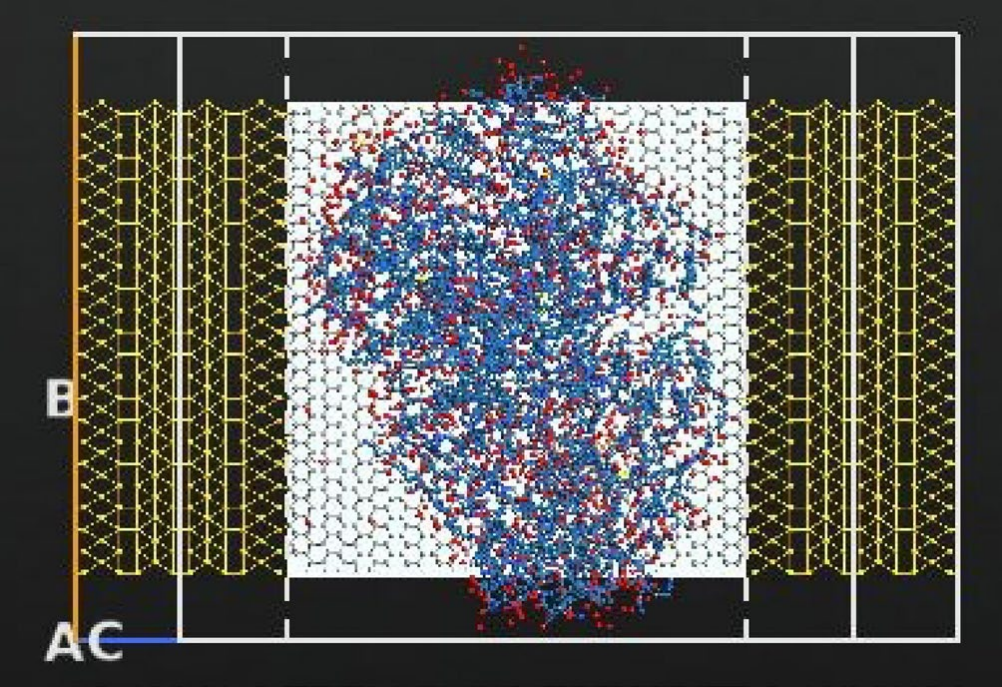
Top view of the designed biosensor with SARS-CoV-2 human neutralizing antibody, Cu MNPs and Rabies virus. Produced by QuantumATK software (version 2018.06).

**Figure 7:**
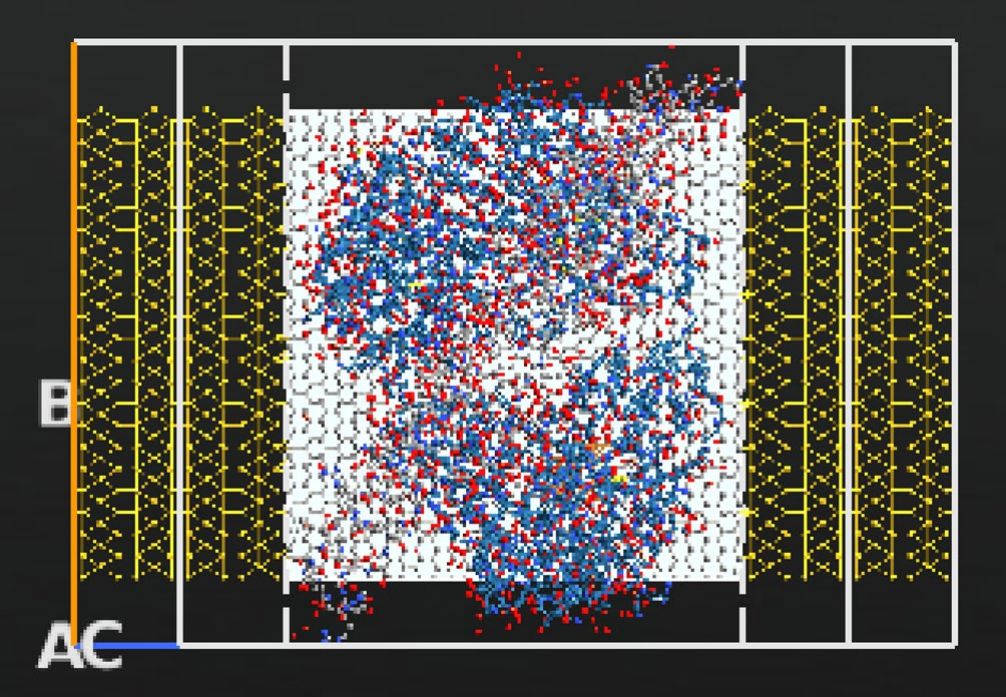
Top view of the designed biosensor with SARS-CoV-2 human neutralizing antibody, Cu MNPs and MERS virus. Produced by QuantumATK software (version 2018.06).

The biosensor’s selectivity was tested against other viruses namely, Rabies and MERS. Both viruses were imported from the same Data Bank with PDB ID’s of 7C20 for Rabies and 4NJL for MERS^75,76^. The reason behind choosing the Rabies as a testing target may be interesting, researches have reported that active and inactive Rabies samples can be utilized as efficient vaccinations against other viruses including COVID-19^77^, hence we wanted to study the biosensor’s behavior towards such a virus. Additionally, Rabies virus has a comparatively small size (~ 800 atoms) when imported into our software, thus cutting off the simulation time as possible. While choosing MERS was mainly because of the several similarities it has with COVID-19, namely, both target the same cellular part which is the Human Angiotensin 1 converting enzyme 2 receptor^78^, and their sequence identities are almost 40% identical^79^. The sensor was coated with bio molecules as sensing probes, in this case anti SARS-CoV-2 human neutralizing antibody was employed with PDB ID 7K8N^80^. The Rabies, MERS viruses, and antibody are shown in Fig. 4. Moreover, the full structure of the biosensor with the testing viruses and antibody is displayed in Figs. 6 and 7. It is worth noting that a low concentration of the bio molecules (i.e., Rabies antigen, MERS antigen, SARS-CoV-2 human neutralizing antibody) was employed here (only one molecule of each) with the intention of testing the sensor’s sensitivity at very low concentrations and speeding up the simulation process. This design is a proof of concept that is anticipated to provide a rapid, on-spot and accurate detection of COVID-19, and it is a road map to follow when proceeding with fabrication.

### Electrode-channel interface

A special plugin in the Quantum ATK software was used to create a stress free (relaxed) interfaces for our FET. In this design we have two interfaces one on each side namely, left and right gold electrodes and rGO channel. To understand the nature of these interfaces their transmission spectrums were generated. It was confirmed that due to the continuous graphene nanoribbons down-sizing the contact resistance can dominate. This was attributed to the decreased number of available conduction modes^81^. However, to reduce this effect, end-contacted electrodes have been employed for the rGO-FET biosensor^82^. Moreover, the interaction nature that occurs between the rGO channel and the metal electrode can go in two directions either physisorption or chemisorption, where the former does not introduce major electronic alteration, the latter does. Hence, gold was utilized since it belongs to the physisorption family^32^.

### Computational method

Single atoms interactions with the aid of the non-equilibrium Green’s function (NEGF) formalism have two approaches namely, ab initio and semi-empirical (SE). Here in our design SE approach was adopted since it has lower computational cost, can be fitted to experimental data and may also offer more accurate results^49,83,84^. In this work, Extended Huckel (EH) parameters accompanied with self-consistent (SC) Hartree potential were used to carry out the system’s study. A 10 Hartree mesh cut-off was fixed along all the device’s calculations also a k-points grid of 3 × 3 × 68 for the Brillouin Zone is used. Moreover, Poisson equation with conditional margins on the electrodes were employed such as, Dirichlet condition along C direction and Neumann boundary condition along A and B directions^85^. A combination of Hoffman and Cerda basis sets were applied on the carbon atoms in the system to reduce the computation time as possible while keeping a good accuracy. Hoffman basis set were applied on all other atoms^85^. The transmission spectrum was calculated in an energy range from − 2 to 2 with 201 sampling points. To calculate the transmission between two electrodes (drain and source) under a certain applied voltage Eq. (3) was used^86,87^:

3 $${\text{T(E,V) = Tr[}}\Gamma _{{\text{L}}} {\text{(E,V)G(E,V)}}\Gamma _{{\text{R}}} {\text{(E,V)G(E,V)]}}$$

where, the left and right electrodes coupling matrices are represented by Г_L_(E,V) and Г_R_(E,V) respectively and G(E,V) is the device’s active region Green’s function. All simulations and studies have been conducted using Quantumwise Atomistix Tool Kit (ATK) with Virtual NanoLab package. The software allows the nano-scale studies by providing a graphical user interface known as Virtual Nano Lab (VNL)^88^. All simulations and runs were carried out with the help of a high-performance computing environment (HPC). In this study, 2 computing nodes each with 36 cores were utilized in all the simulations.

## Results and discussions

### Channel’s performance

**Figure 8:**
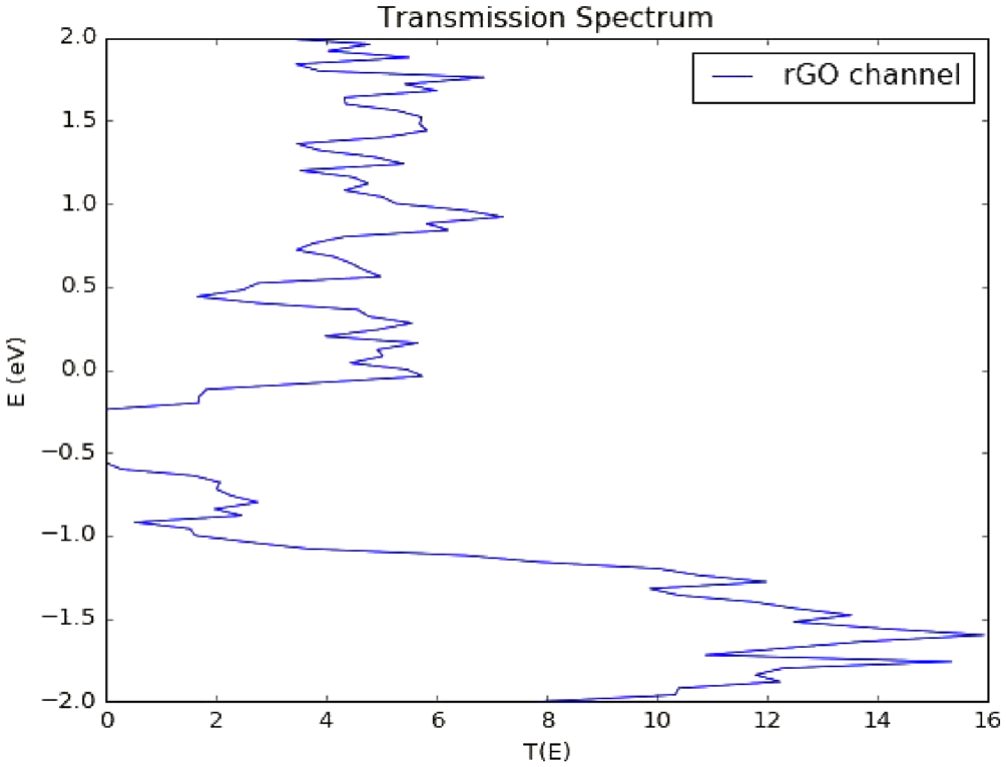
rGO channel's transmission spectrum. Produced by QuantumATK software (version 2018.06).

**Figure 9:**
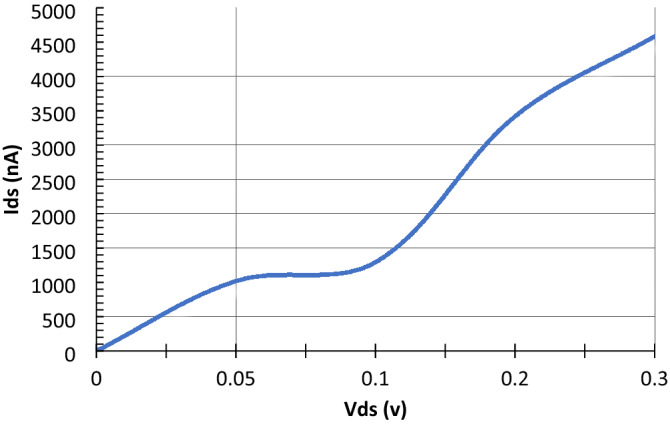
I–V curve of the bare G-FET sensor at V_g_ = 1 V. Produced by Excel based on the data generated from QuantumATK software.

**Figure 10:**
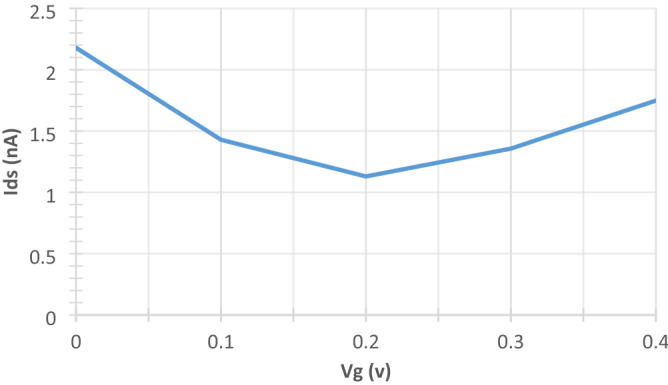
Transfer curve of the G-FET sensor at V_ds_ = 0.05 V. Produced by Excel based on the data generated from QuantumATK software.

The channel semiconducting nature was further confirmed from its zero bias transmission spectrum. It is evident from the rGO channel’s transmission spectrum in Fig. 8 that it has semiconducting properties due to the obvious 0.4 transmission bandgap below the fermi level (E_f_ = 6 eV). The Fermi level value is in a good agreement to what is reported in the literature varying from 4.2 to 6.8 eV depending on the amount and type of oxygen functional groups within the sample^89–91^. It can also be observed that the figure is asymmetric in the positive and negative regimes. This is because of the unique structure of the rGO which makes it non uniform since the oxygen functional groups are randomly scattered within it. This randomness can also cause an increased back scattering leading to a suppressed conductance^92^. Specifically, epoxies are the main reason of the broken symmetry between the holes and electrons close to the Dirac point^93^. Moreover, the covalent bonds between the graphene and the oxygen functional groups have a noticeable impact on the transmission spectrum which is apparent as conductance dips in the graph. It was proved that the sp^2^ hybridization can be transformed into sp^3^ hybridization as a result of covalent functionalization however this approach offers higher stability^92,94^. Additionally, it can be noticed that there is a suppression in the valence band compared to the conduction band. This was attributed to the oxygen functional groups near the graphene ribbon edges since their near-edge location leads to magnify the back scattering^92^. This suppression is expected to significantly increase when adding the metal electrodes owing to the interface’s contact resistance.rGO FET biosensor characterization. The output (I_ds_ − V_ds_) and transfer (I_ds_ − V_g_) curves of the bare sensor are generated. It can be observed from the below output characteristic (Fig. 9) that a linear relation in the biasing voltage range from 0 to 0.3 V at a gate voltage of 1 V is evidential. Figure 8 shows that an ohmic contact between the rGO channel and the gold electrodes is obtained. Moreover, the drain source voltage was fixed at 0.05 V while sweeping the gate voltage between 0 and 0.4 V, the resulting transfer curve is shown in Fig. 10. The curve exhibits an obvious v-shaped, ambipolar field effect transistor trend. The voltage at the minimum current value known as Dirac point is almost 0.2 V, this slightly positive shift in the Dirac point was attributed to some trapped impurities in the SiO_2_ wafer below the rGO channel which causes intrinsic p-doping impact on the graphene^91,95^.

### Biosensor’s transmission spectrum

**Figure 11:**
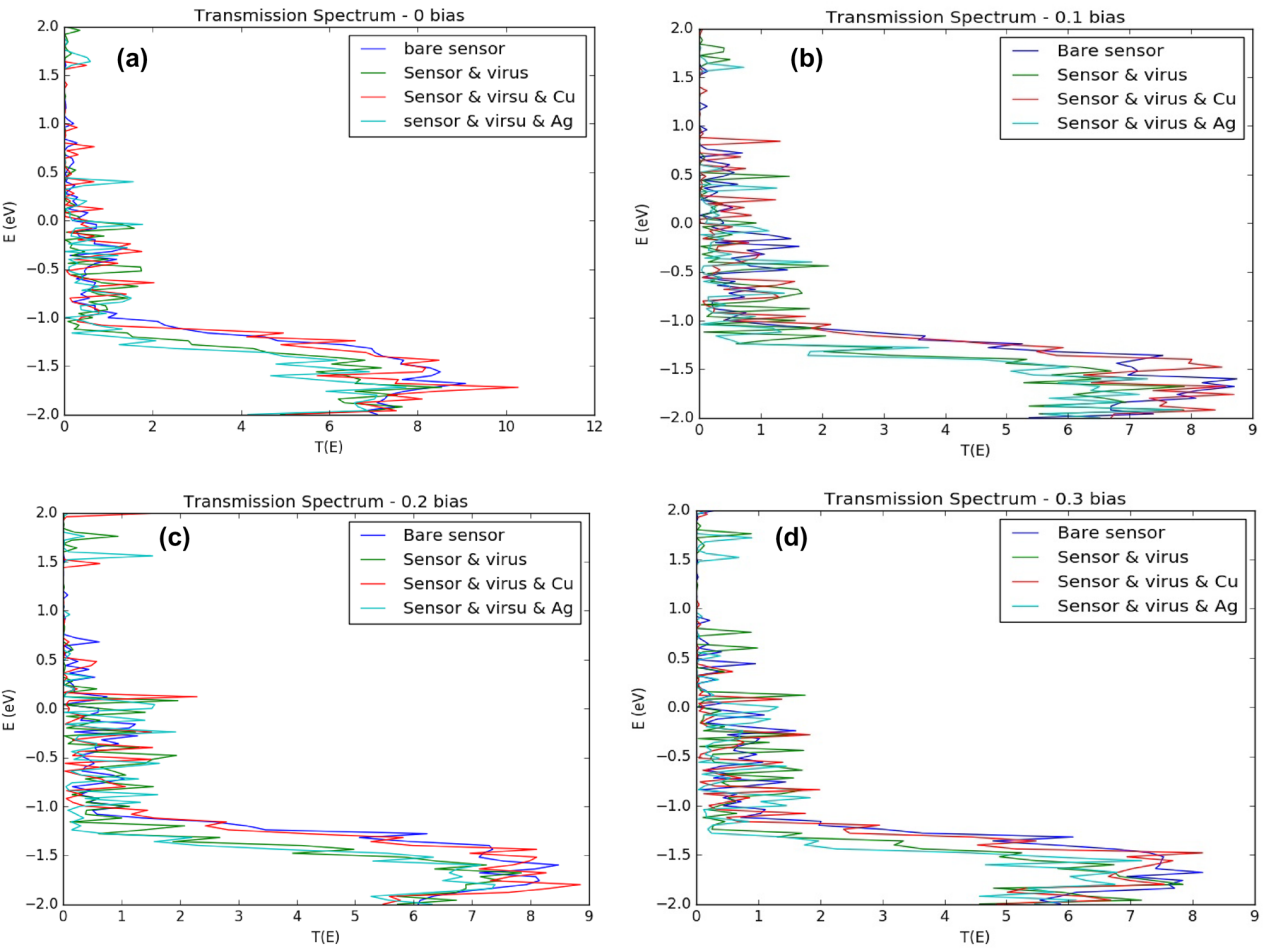
Transmission spectrum of the bare (without antibodies and MNP’s), virus bound, and MNPs decorated sensor at (**a**) V_bias_ = 0.0 V (**b**) V_bias_ = 0.1 V (**c**) V_bias_ = 0.2 V (**d**) V_bias_ = 0.3 V. Produced by QuantumATK software (version 2018.06).

Transmission spectrum figures for the bare, virus, MNP’s, and virus decorated sensor were produced for the bias voltages (V_ds_) of 0.1, 0.2 and 0.3 V. The spectrums are shown in Fig. 11 below. At first glance, it can be seen that all of the spectrums exhibit an overall similar behavior that is suppressed valence band, multiple nonsymmetrical oscillations taking over most of the schemes, reduced transmission and diminishing, and moveable transport gap. Additionally, looking more closely at the conduction band peak amplitude, we can notice that it slowly decreases with increasing applied voltage as follows, from 9 to 8 for bare sensor, from 8.6 to 8.1 for the virus-bound sensor, from 10.3 to 8.1 for the Cu MNPs decorated virus-bound sensor and from 7.9 to 7.2 for the Ag MNPs decorated virus-bound sensor. The transmission spectra for silver nanoparticles is higher than copper nanoparticles because of the different optical properties of the two metals. Silver has a higher electrical conductivity than copper because it has a higher number of free electrons available to carry electrical current. Furthermore, Silver has a lower resistivity than copper, this means that it offers less resistance to the flow of electrons, leading to a higher conductivity. In addition, silver nanoparticles have a stronger surface plasmon resonance (SPR) than copper nanoparticles^96,97^. We have previously expected that transmission spectrum of the whole system will have a significant suppression in its valence band until reaching a full suppression in some positions. This demeanor can mainly be attributed to two main reasons: first, the near-edge positions of the functional oxygen groups which magnifies the back scattering and states’ localization^92^, and second, the addition of the gold electrodes or metal contacts (MC) that introduces an added contact resistance resulting in a decreased conductance. Contact resistance is actually one of the biggest challenges when designing a device based on a 2D material since a great portion of the applied bias is wasted by the parasitic resistance^81^. This phenomenon which is known as metallization can easily be spotted here. Regardless of the rising bias voltage there is no noticeable increase in transmission. Metallization effect caused by the gold MCs also introduces additional three major observations in our readings, primarily, the several little peaks that are apparent in each reading are normally a direct outcome of metallization. These peaks are known as Lorentzians^81^. According to Lorentz the force between an electron and atom’s nucleus follows Hooke’s law, also known as spring force. The existence of such force obliges the electrons to bear multiple oscillations caused by the electric field changes^98^. These oscillations are seen here as several little peaks. Secondly, metallization can also be observed from the unmistakably reduced transmission compared to the channel’s before adding the MCs (from 16 to almost 9). The occurrence of Lorentzians and reduced conductance has been specifically attributed to the destructive interference impact on the travelling electrons along the FET which is the result of the constant broadening nature of the MCs^99^. Finally, transport gap is also affected by the MCs, as can be concluded from the spectrums, the gap has diminished, got significantly smaller (transmission less than 0.1) or changed its position in most of the cases. Poljak’s group has related this manner to the rGO ribbon dimensions. They have concluded that the transport gap is strongly affected by the graphene ribbon width and length, while increasing the former leads to diminished gap, stretching the latter results in a broader one^81^. Apparently, from our graphs it is obvious that the channel’s width impact has dominated. Although the produced transmission figures reveal a similar pattern in general, they are also different, each one of them is unique and shows a singular response which will naturally lead to distinct current value for every case.

### Biosensor’s output curves

**Figure 12:**
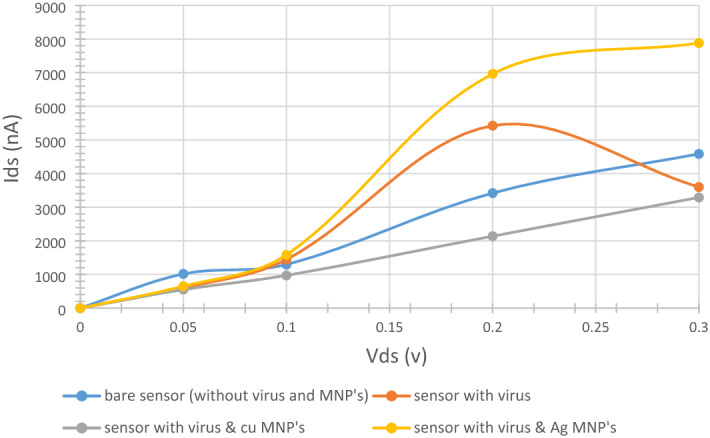
I-V curves of bare (without antibodies and MNP’s), virus-bound and MNPs decorated sensor at V_g_ = 1 V. Produced by Excel based on the data generated from QuantumATK software.

The biosensor’s bias voltage (V_ds_) versus the channel’s current (I_ds_) were plotted at a fixed gate voltage (V_g_) of 1 V to further study the sensor’s performance. Figure 12 depicts the output curves of bare sensor, sensor with virus, and sensor decorated with MNPs and virus. It is noticeable that the bare and virus and MNPs (Cu and Ag) functionalized cases show a linear relationship indicating an ohmic contact between the MCs and the rGO channel^12,19^. On the contrary, the virus bound sensor shows a distinct negative differential resistance (NDR) behavior, this result was anticipated for the reason that, MNPs’ channel decoration was employed here to avoid the direct contact between the bio-target and the rGO channel since that can lead to altering the electronic structure of the rGO which eventually result in a distorted electrons distribution^64^. In Serhan Y.’s work, the NDR phenomenon was also linked to the coverage ratio, which is the percentage of carbon atoms having bonds with Oxygen functional groups in a specimen. He has concluded that rGO sheets with coverage ratios of 6.25 and 12.5% exhibit NDR behavior^100^. It is worth mentioning that our rGO channel has a coverage ratio of 11.2% which is very close to the ratios in the mentioned research. Obviously, functionalizing the rGO channel with MNPs has helped to eliminate NDR effect and enhance the sensor’s bio-sensing performance. This definitely can be added to the list of advantages for using MNPs. Moreover, introducing Cu and Ag nanoparticles has firmly affected the sensor’s I–V curve, the bare sensor had a current range between 1.293 and 4.585 μA at V_ds_ ranging from 0 to 0.3 V and V_g_ of 1 V (blue line) in the graph, while utilizing Cu atoms has lowered this range from 0.9713 to 3.290 μA (grey line) with same previous biasing and gate voltages conditions. Adding Ag atoms has unquestionably raised it to be from 1.581 to 7.880 μA (yellow line) keeping the same V_ds_ and V_g_. These opposing trends were attributed to the charge carriers’ movement between the introduced MNPs and the rGO sheet. It can be noticed that the movement was from the device’s channel to the MNPs in the Cu atoms case while it was in the opposite direction in the Ag atoms situation. MNPs can either be acceptors (Cu in our case) or donors (Ag here) of charge carriers affecting the current value in the device^101^. These dramatic changes in the output curve from the reference case (bare sensor) can absolutely be used as a detection signal of our bio-target. Another important metric that can be used to measure the sensor’s sensitivity is the current variation ($$\Delta I)$$. Current variations is calculated as the difference between the drain current of the sensor without virus ($${{I}_{ds}}_{before})$$ and the drain current of the sensor with virus ($${{I}_{ds}}_{after})$$^102^ as shown in Eq. (4) below:

4 $$\Delta I={Ids}_{before}- {Ids}_{after}$$

**Figure 13:**
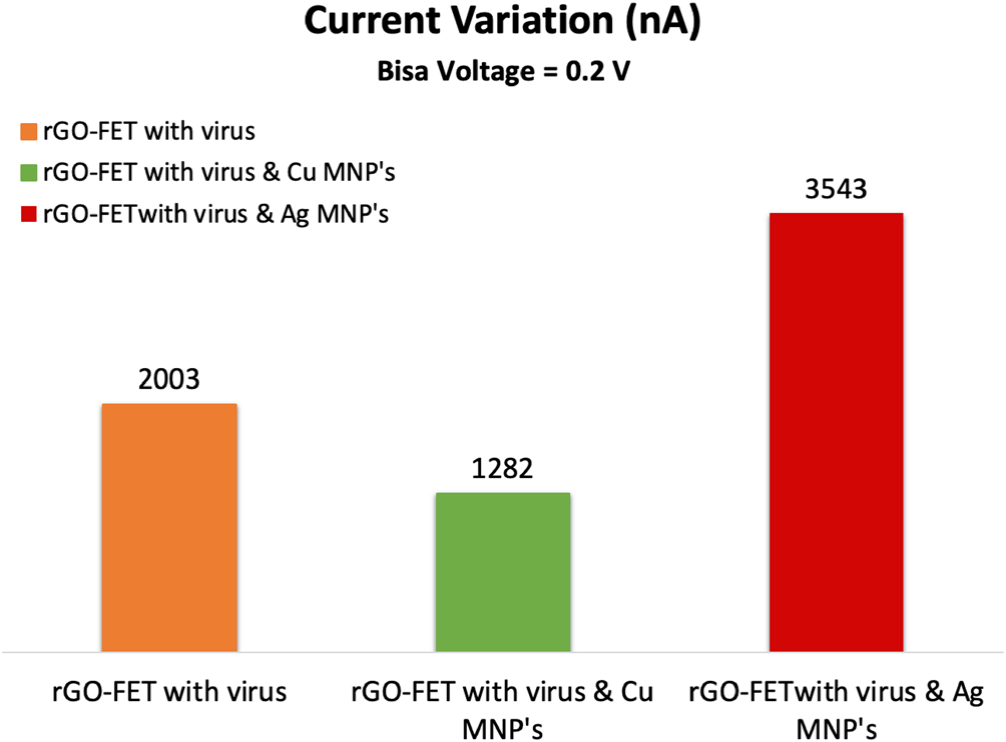
Current variation of the sensor due to the addition of the target molecule (COVID-19 spike antigen). Produced by Excel based on the data generated from QuantumATK software.

Figure 13 depicts the current variations at different sensor’s states. It can be seen that the current variation was the highest in the case of Ag MNP’s decorated sensor. This leads to a very important conclusion which is, the silver nanoparticles shows the best performance and selectivity, since it introduces the largest variation in the drain current which is ${\Delta I}_{Ag}=3543$^103^ . This exceptional behavior created by the Ag MNP’s can be attributed to its interaction nature with the rGO channel as they act as charge carriers donors which eventually lead to electrical current increment^101^ and higher sensitivity.

### Biosensor’s selectivity

**Figure 14:**
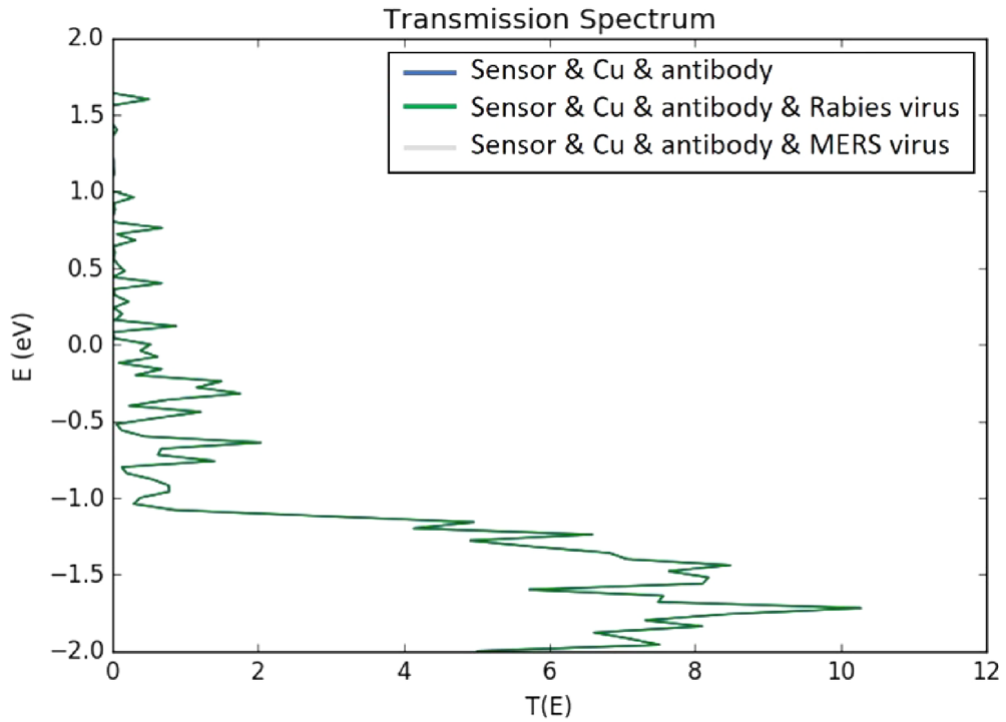
Transmission spectrums of SARS-CoV-2 human neutralizing antibody decorated sensors before and after addition of Rabies and MERS viruses. Produced by QuantumATK software (version 2018.06).

The sensor’s specificity against other viruses such as Rabies and MERS viruses was also tested. It can be noticed from Fig. 14 below that no detectable change was spotted on the transmission spectrum by the software since both Rabies and MERS viruses did not bind to the SARS-CoV-2 spike antibody. The spectrums here follow the same previously discussed trend with suppressed valence band and transmission reaching to maximum of 10. The detection of COVID-19 virus via the proposed rGO-FET is expected to be rapid and on real time in experiment since the sensor current varies once the virus is placed on the sensors’ channel. The adsorption of the target molecule (COVID-19 antigen) occurs immediately leading to a specific current variation^104,105^. The accuracy of the sensor is confirmed as the sensor showed a clear variation in its electronic properties such as current due to the addition of COVID-19 antigen. However, the addition of Rabies and MERS viruses did not modify the sensor’s readings.

## Conclusion

In this work, we designed, studied and simulated an rGO field effect transistor based biosensor to rapidly and accurately detect SARS-CoV-2 virus. The rGO channel, which was the detection site, was passivated with hydrogen at the edges. Moreover, it was decorated with metallic nanoparticles (copper and silver) and functionalized with SARS-CoV-2 human neutralizing antibody. The rGO sheet was connected to gold electrodes to finally form the FET based biosensor. The whole arrangement was then tested using non-equilibrium Green’s function method along with semiempirical approach namely, extended Huckle. The self-consistent calculations revealed that the sensor is able to exhibit distinct results in each case. Various electronic transport characteristics such as: current, transmission spectrums and output curves were studied for the biosensor before and after viral exposure situations. The variations in the electronic characteristics due to the addition of the target molecule can be used as the bio-target detection signals. Additionally, the sensor’s selectivity was also tested against Rabies virus which produced the same transmission spectrum of the sensor before adding Rabies virus. However, the fabrication process of such bio-sensors is expected to face some challenges in manufacturing of end contacted electrodes since it can be time consuming and complex process. Moreover, choosing the suitable molecular precursors to control the graphene nanoribbons structure is not an easy task. Additionally, rGO unified mass production has always been a challenge, since each sample of rGO has a unique electronic structure because oxygen functional groups scatters randomly on the graphene surface and finally, the usage of real viral samples may affect the biosensor’s sensitivity since it may cause many non-specific interactions. In conclusion, this work is expected to aid in the on-going SARS-CoV-2 worldwide pandemic as it proposes an efficient, rapid, cost effective, and precise viral detection alternative.

## Data Availability

All data generated or analyzed during this study are included in this published article.
